# Closing the Digital Divide in Interventions for Substance Use Disorder

**DOI:** 10.20900/jpbs.20240002

**Published:** 2024-03-26

**Authors:** Jazmin Hampton, Purity Mugambi, Emily Caggiano, Reynalde Eugene, Alycia Valente, Melissa Taylor, Stephanie Carreiro

**Affiliations:** 1 Division of Toxicology, Department of Emergency Medicine, University of Massachusetts Chan Medical School, Worcester, MA 01655, USA; 2 Washington University of Health and Science, San Pedro, Belize, Central America; 3 Division of Public Health, Walden University, Minneapolis, MN 55401, USA; 4 Manning College of Information and Computer Sciences, University of Massachusetts-Amherst, Amherst, MA 01003, USA

**Keywords:** substance use disorder, digital health, mHealth, social determinants of health, digital inequities, digital divide, machine learning, artificial intelligence, algorithmic bias

## Abstract

Digital health interventions are exploding in today’s medical practice and have tremendous potential to support the treatment of substance use disorders (SUD). Developers and healthcare providers alike must be cognizant of the potential for digital interventions to exacerbate existing inequities in SUD treatment, particularly as they relate to Social Determinants of Health (SDoH). To explore this evolving area of study, this manuscript will review the existing concepts of the digital divide and digital inequities, and the role SDoH play as drivers of digital inequities. We will then explore how the data used and modeling strategies can create bias in digital health tools for SUD. Finally, we will discuss potential solutions and future directions to bridge these gaps including smartphone ownership, Wi-Fi access, digital literacy, and mitigation of historical, algorithmic, and measurement bias. Thoughtful design of digital interventions is quintessential to reduce the risk of bias, decrease the digital divide, and create equitable health outcomes for individuals with SUD.

## INTRODUCTION

Digital health interventions, or those that leverage computing platforms, connectivity, software, and sensors for health care and related uses [1], are rapidly growing within the field of medicine. As devices such as smartphones, wearable devices, and computers have become ubiquitous in patients’ lives, they can be used to collect tremendous amounts of data, rapidly analyze trends, facilitate communication, and deliver interventions. For example, smart phones applications (or “apps”) exist that can serve as a portal to a telehealth visit, a source of information to learn about one’s diagnosis or a means to locate critically needed services. Wearable sensors, such as smartwatches have the ability to collect continuous physiologic data, which can be synthesized into digital biomarkers that predict disease or outcomes.

In a national survey conducted by the AMA including a mix of primary care physicians and specialists, opinions that digital health tools were advantageous to patient care increased from 85% in 2016 to 93% in 2022. The average number of digital health tools used by a physician growing from 2.2 to 3.8 over that same time period [2]. In a recent cross-sectional study of United States (US) accountable care organizations by Miller–Rosales et al., approximately one-third of the organizations surveyed integrated at least one digital health technology at the system level to support treatment of opioid use disorder (OUD) [3]. Overall, digital health technologies are being used to improve access, increase quality, personalize medicine, and reduce both costs and inefficiencies [1]. However, vulnerable populations face a variety of barriers that contribute to unequal access of digital health interventions and their related benefits [4]. Quality improvements on healthcare disparities have been consistently documented to improve the treatment, diagnosis, and health outcomes of a variety of conditions [5]. Therefore, in evaluating the potential of digital health interventions to help bolster current SUD treatment efforts, it is important to consider the factors that contribute to potential bias.

Addressing the inequality of digital health interventions is of particular interest related to their implementation for substance use disorder (SUD). The SUD crisis continues to devastate the US, with drug overdose deaths climbing from approximately 72,000 in 2019 to 107,000 in 2022 [6]. Recent reports show that 16.5% of Americans aged 12 and older met DSM-V criteria for a diagnosis of a SUD in 2022, and it is estimated that less than 20% of people with SUD receive treatment [7,8]. Other intersectional variables including socioeconomic status and race highlight differential risk for people with SUD. For example, people experiencing homelessness have more difficulty accessing treatment, and higher risk of overdose death [9]. Individuals who identify as black, indigenous, and people of color (BIPOC) have disproportionate levels of SUD, fewer treatment options, and have more negative health outcomes which are compounded by systemic racism and stigma [10,11].

Digital health interventions have been proposed as solutions to some of the barriers to SUD treatment [12,13], but have the potential to inadvertently worsen disparities if not developed and deployed with careful attention to the barriers that target end-users already face. The current manuscript seeks to further explore the potential pitfalls, but also the promise, of digital health interventions for SUD through a health equity lens. We will highlight key concepts related to digital inequities specific to the case of SUD, SDoH as drivers of digital inequities, and ways in which data and algorithms contribute to the disparities of digital health interventions within this population. Finally, we will discuss solutions and relevant future directions.

## SOCIAL DETERMINANTS OF HEALTH AS DRIVERS OF DIGITAL INEQUITIES

Social determinants of health are broadly defined by the Centers for Disease Control (CDC) as “conditions in the places where people live, learn, work, and play that affect a wide range of health risks and outcomes” [14]. These are subdivided into five domain areas including economic stability, neighborhood, education access and quality, health access and quality, and social and cultural context. Broadband access and digital health literacy have been coined ‘super determinants of health’ because of their significant influences on health outcomes [15]. Digital inequities, defined as differential use of digital solutions based on demographic and socioeconomic characteristics, limit the promise of mobile health (mHealth) and further divide the US population in terms of healthcare access and outcomes. The related term “digital divide” refers to the disparity of technological applications that stem predominantly from issues with access and to a lesser degree usage [16]. While the digital space now provides more access to care for those that need it, getting access to those digital spaces still remains a challenge. Lack of digital equipment, access, and knowledge to use digital resources all fuel the divide [17]. Specifically for SUD, digital inequity present a major barrier to mHealth interventions as it also threatens sobriety and recovery.

The rapid dissemination and uptake of telehealth in recent years (throughout a variety of medical specialties, including addiction medicine [4]), driven by the COVID-19 pandemic, has provided some insight into barriers of digital health implementation. In the addiction medicine space, telehealth facilitated services such as virtual peer support meetings (e.g., Alcoholics Anonymous), and virtual prescribing of medications for addiction treatment (buprenorphine and methadone) [4]. Widespread reliance on telehealth created a unique opportunity to expose both the promise of digital health and the potential inequities these tools exacerbate. Four important areas of concern are broadband access, digital device access, privacy, and digital literacy.

### Broadband Access

The lack of equal access to broadband throughout the country has led to disparities in health care delivery, health literacy and public health messaging [18]. Areas of limited or no connectivity are mostly in rural and select urban communities, and disproportionately affect those of lower socioeconomic status, people over the age of 65, and communities who identify as BIPOC [15]. Connectivity has repeatedly been linked to better health outcomes: for example, in a study of 3026 participants with OUD, it was found that both cell phone and internet use was associated with increased days of medication for opioid use disorder (MOUD) [19]. The COVID-19 pandemic increased society’s dependence on broadband, and reliable internet access has become necessary to link people to jobs, education, information, and healthcare. The digital divide has direct health consequences with those living in areas with limited internet access, creating less access to primary care, higher rates of chronic disease, and more preventable hospitalizations [20]. Individuals with SUD experience stigma and marginalization at baseline [11], and decreased internet access limits economic, education, and treatment options and compounds existing health disparities.

### Digital Device Access

With a smartphone being the most common tool from which to launch digital interventions, smartphone ownership is another key factor. Within the SUD literature, reported smartphone usage varies from 57% to 94% [21–23]. For example, in a study of 178 patients receiving methadone maintenance treatment, 94% noted that they owned a smartphone within the past year [22]. However, it is important to consider disparities in the quality of smartphone technology when considering the larger picture of the digital divide. Many studies that assess phone ownership do not differentiate traditional smartphones from feature phones, the latter of which have internet access but lack the advanced functionality of a true smartphone [24]. Lower-end processing power in feature phones significantly impact the performance of, and user experience with, digital interventions. The Android Go operating system for example, developed and distributed by Google, has a subset of the features that the complete Android operating system contains, resulting in a stripped-down version requiring less processing power, storage, and memory to operate and run applications [25]. Data is not available regarding the digital capabilities of smartphones used by people with SUD; however, in our teams’ experience feature phones are more common than in the general population. Developers of digital interventions for SUD will need to consider whether (and how) to support lower-end smartphones with their application development strategies.

An additional consideration once patients have access to the hardware to use digital health interventions is the access to the software itself if there are associated fees. Currently many digital health tools for SUD are available only through research studies. However, the number of prescription digital therapeutics is growing, raising questions regarding payer reimbursement. Uninsured or underinsured patients would again be in a position of disparity; this will be an area to monitor closely as policies evolve.

### Privacy and Cultural Concerns

Besides access issues related to smartphone ownership and quality, there are a variety of patient viewpoints that affect usage of digital interventions for SUD treatment. In a recent review article of 22 studies looking at digital interventions, overall acceptability of mHealth interventions for SUD was found to be high [26]. However, some studies have revealed specific concerns from end-users. For example, usage of geolocation has been repeatedly associated with poor acceptance [27]. One study cited that 46% of survey respondents receiving addiction treatment rated the use of geolocation as unacceptable. An earlier study, sampling patients in the emergency department being treated for drug and alcohol use, noted that those suffering from drug misuse were less likely to be accepting of technological-based solutions for SUD information. The authors cited a high rate (54%) of concern for confidentiality (the protection of personal information) among this population and concluded that increased clarification surrounding privacy policies may be useful to alleviate this concern [27]. This concern for privacy was also illustrated in a study looking at mHealth acceptance in China, where the authors suggested that strong cultural stigma and fear for repercussions drove negative perceptions of technology usage [28]. Although privacy is always a concern when monitoring individual level data, this issue is amplified by the stigmatized nature of the disease process and the potential consequences (legal and otherwise) of exposure. Other key ideas related to differential usage in the literature include age, with subject populations often being skewed towards younger groups, and cultural differences affecting overall acceptability and uptake [26].

### Digital Literacy

Perhaps the least well-studied barrier related to SDoH is digital literacy, or the ability to find, evaluate, and communicate information using digital platforms, which is required to use digital health tools to their full capacity [29]. Just as overall health literacy has been positively correlated with improved preventive behaviors and health outcomes, digital literacy similarly empowers individuals to understand, meaningfully engage with, and apply content provided [30]. The converse is also true, and the lack of digital health literacy makes it difficult or impossible to leverage the power of digital health interventions.

## ALGORITHMS AS SOURCES OF DISPARITIES

The increased adoption of digital devices in medicine has increased data collection, and consequently the use of algorithms to learn insights from that data. Machine learning (ML) is a subfield of artificial intelligence in which statistical techniques are utilized to learn patterns in data without a computer programmer explicitly providing the instructions to the computer [31–32]. Machine learning has gained popularity in medicine and in SUD treatment and is commonly used in digital interventions. While ML algorithms have the power to reveal new knowledge to us, they also can replicate and magnify existing disparities as a consequence of the data used in learning as well as the algorithmic choices made when training the models.

Historical, label, and measurement biases are some of the main ways systemic and human prejudices can be introduced into ML models and propagated by them. Historical bias [33–36] arises when data collected in the past, which may contain human and systemic prejudices and stereotypes, is used to learn a function that is then used to predict the future. Despite the modeler’s best efforts, the generated model will represent “the world as it is or was” [37], that is, circumstances when the data was originally collected. Label bias [38] is a subtype of historical bias and refers to the high likelihood of minoritized groups being assigned incorrect outcomes when learning the models. For example, underrating and undertreatment of pain has been well-documented for certain patient groups, including women, racial minorities, and people with SUD [39–42]. Using data collected in the past to build a model that predicts the need for analgesia would perform poorly for these categories of patients despite achieving high accuracy in the training dataset. This bias severely affects supervised learning, a subfield of ML that learns from data with assigned outcomes and is the subfield of ML most implemented in medicine [43–45] and SUD [46–47]. Measurement bias refers to the under or overrepresentation of certain groups during data collection. For instance, racial minorities in the US could be over-represented in criminal data [48] as a consequence of being overpoliced or white males could be overrepresented in data used to train facial recognition software [49]. In SUD, measurement bias can be of concern due to lack of access to digital tools and consistency in their use. Since both factors are strongly affected by SDoH, digital inequities could result in some populations being underrepresented and therefore not included in the learned models. For example: studies requiring participants to have their own mobile devices can lead to a biased sample of participants, resulting in a model that has minimal data on the most at-risk members of the target population [38,50].

Model design choices also have the potential to introduce biases, including model architecture, optimization (approach for reducing the errors when learning a model), hyperparameters (parameters that specify the details of the learning a model), and loss functions (approaches to compute the error a model is making). This is referred to as algorithmic bias [34,51]. For instance, many models in supervised learning have one objective; to reduce the error in assigning the correct label to every data row. Prioritizing one objective, in this case accuracy on the held-out dataset, inevitably introduces new trade-offs [51]. The growing field of fairness in ML has shown that models can trade off fairness for high accuracy on the held-out dataset [52–54]. In other words, the model with high accuracy can perform substantially worse on underrepresented groups. This bias is exacerbated when the group that is underrepresented in the data is then over-represented in the real world.

Digital devices have also proliferated the collection and use of digital biomarkers, or end-user-generated markers derived from these technologies which indicate normal physiology, pathology, or response to treatment [55,56]. In addition to suffering from the listed challenges, the performance of digital biomarkers may differ across patient groups due to variations in sensor data. For instance, it has been shown that pulse oximeters have a significant decrease in accuracy for darker-skinned people compared to those with lighter skin tones [57] and that accuracy of heart rate variability can vary with skin tone in some sensors [58]. If the devices are poorly calibrated for particular patient groups, digital biomarkers would still be inaccurate despite other efforts such as ensuring equity in representation, consistency in the use of the devices, and using proper modeling approaches.

## POTENTIAL SOLUTIONS AND FUTURE DIRECTIONS

In addition to providing technological innovation, digital interventions for SUD must also help overcome existing barriers and challenges as opposed to exacerbating them. To ensure these promising tools reach their full potential, those who need them most need to also have access (to the necessary broadband and devices), skills (digital literacy), and acceptance to unbiased tools that work for them. To achieve this laudable goal, researchers, developers, clinicians, and policymakers need to take steps to address all of these components.

### Increasing Broadband Access

Programs to increase internet connectivity of all communities are at the heart of decreasing the digital divide. According to the 2020 broadband report, 22.3% of American rural areas and 27.7% of American Tribal Lands still lack high-speed broadband internet coverage. The United States Food and Drug Administration (FDA) launched the ReConnect Loan and Grant Program in 2018 which invested over $1 billion to expand high-speed broadband infrastructure in underserved rural areas and Tribal Lands. Local grassroots endeavors have highlighted the need for broadband on the state level, and potential bills such as the H.R.1783 - Accessible, Affordable Internet for All Act, work to give municipalities the funds to increase digital equity. The FCC has launched the Connect2Health Taskforce, which is charged with creating a digital analytic platform to map and study the connection between broadband access and health in key focus areas, including opioid and substance use [20]. The United States Department of Agriculture (USDA) and FCC should continue to support and expand the efforts to improve high-speed internet access, research, and equity through programs like ReConnect and Connect2Health. Other less costly solutions to Wi-Fi access among the SUD population include creating safe facilities with free Wi-Fi that can be used for treatment-related telehealth visits.

Historically, funded research has favored treatment and biological factors of SUD, leaving social factors relatively understudied [10]. More data are needed to identify factors that may positively or negatively affect the equitable application of digital health interventions in the SUD population. Steps to increase awareness of these historical biases which are likely embedded in the datasets collected during modeling should be a priority. Researchers being well-informed of the different aspects and level of obstacles that underserved communities face to access health care allows us to better focus on possible solutions to the digital divide. Digital tools can also prompt healthcare providers to assess and consider SDoH to identify patient needs. One such example is RAE cHealth, a digital intervention for people in recovery from SUD which consists of a wearable device and a mobile app [59]. The system couples digital biomarkers of stress and drug craving with structured needs assessments of SDoH to objectively identify and remedy barriers to SUD recovery.

### Increasing Digital Device Access

Expanding ownership of smartphones is a key initial step that is already underway. There are established programs providing smartphones and services to people in need, however these programs often require a mailing address. Literature on people with SUD has highlighted a relationship between SUD, homelessness, low-income, and low smartphone ownership. For that reason, programs offered by the Federal Communications Commission (FCC) to address financial barriers to mobile phone use among low-income populations, such as the Lifeline program, should consider broadening the requirements to include and increase access to mobile phones for people with SUD who are also experiencing homelessness. A study on mobile phone use in older adults experiencing homelessness found that among people diagnosed with a SUD who had a mobile phone 53.1% reported having their phone stolen and 31.6% did not have a place to charge their phones [60]. Initiatives to increase access to smartphones could be paired with installation of charging stations in public places. Non-governmental groups such as the telecommunication industry could also explore the idea of funding programs that can increase the use of their services and support underserved populations.

### Avoiding Algorithmic Bias

Careful attention to data curation processes and thoughtful model building can help reduce algorithmic bias. Efforts must be made to diversify data sampling and preprocessing with the aim of balancing representation among groups where possible and appropriate. Evaluating algorithmic models across various performance metrics besides accuracy is needed to reduce bias in developed models. Algorithmic bias could also be reduced by building and evaluating various models using different optimization techniques and hyperparameters. Using datasets obtained through community-based participatory research can reduce biases and increase equity. Furthermore, accounting for SDoH in predictive ML algorithms can help understand complex relationships and tailor treatment approaches, given special attention is made to assure the training data is free of bias that could further marginalize BIPOC and other at-risk communities.

### Addressing Privacy and Cultural Concerns

Acceptability and usability of digital health interventions are dynamic targets that shift with current culture. For example, the COVID-19 pandemic drove the movement for telehealth, which has become familiar and widely acceptable to both patients and clinicians. Non-telehealth digital interventions have benefitted from this movement as well. Many mobile applications on the market are used for self-care and are now incorporated into clinical care for patients [46]. Although digital health interventions to support the care and treatment plan of people with SUD are generally considered acceptable, user privacy remains a topic of concern. Detailed but clear privacy policies may help alleviate this concern. Application developers could make features like geolocation tracking optional, empowering users to make informed decisions about data sharing based on their personal values and comfort. To reduce the risk of an application being too difficult for its target population, app developers may consider using formats and features that are already familiar (e.g. popular social media platforms and web browsers) and incorporating key stakeholders into the design process. Bosse et al., found that patients’ input on the design of treatment apps helps improve treatment experience [61]. The goal for any digital health tool should be simple and seamless engagement because users quickly lose motivation if technical glitches occur [62].

### Improving Digital Literacy

On the individual level, we can assist users by developing educational models that are built within the mobile application. With doing so, users will to be able to jump right in using one space for not only learning how to use the application but using the application itself. On the larger scale, digital health literacy can be improved through public initiatives, particularly in schools. The United States Department of Education, Office of Carrier, Technical, and Adult Education currently funds digital initiatives for learners to be able to navigate technology and leverage learning outside of the classroom. These resources are continuously updated as the digital space expands.

## CONCLUSIONS

Digital health interventions are being widely evaluated in SUD and have generally shown positive effects on SUD recovery metrics [26]. These digital health interventions have tremendous potential to support the treatment of SUD, but also the potential to perpetuate biases if not handled with caution. Developers and healthcare providers alike must be cognizant of the potential for digital interventions to exacerbate existing inequities in SUD treatment, particularly as they relate to SDoH. Thoughtful design of digital interventions is quintessential to reduce the risk of bias, decrease the digital divide, and create better health outcomes for individuals with SUD.

## Data Availability

No data were generated from this manuscript.

## ABBREVIATIONS

US: United States
OUD: opioid use disorder
SUD: substance abuse disorder
SDoH: social determinants of health
CDC: Centers for Disease Control
MOUD: Medications for Opioid Use Disorder
ML: machine learning
FCC: Federal Communication Commission
FDA: United States Food and Drug Administration
USDA: United States Department of Agriculture
BIPOC: Black Indigenous and other People of Color
